# Combined Antiplatelet Therapy Reduces the Proinflammatory Properties of Activated Platelets

**DOI:** 10.1055/a-1682-3415

**Published:** 2021-10-28

**Authors:** Alexandra C.A. Heinzmann, Daniëlle M. Coenen, Tanja Vajen, Judith M.E.M. Cosemans, Rory R. Koenen

**Affiliations:** 1 Department of Biochemistry, Cardiovascular Research Institute Maastricht (CARIM), Maastricht University, Maastricht, the Netherlands; 2 Department of Molecular and Cellular Biochemistry, University of Kentucky College of Medicine, Lexington, Kentucky, United States; 3 Cardiovascular Research Laboratory, Division of Cardiology, Pulmonology and Vascular Medicine, Medical Faculty, Heinrich-Heine-University, Düsseldorf, Germany

**Keywords:** platelets, chemokines, inflammation, monocytes, atherosclerosis, antiplatelet agents

## Abstract

The cause of atherothrombosis is rupture or erosion of atherosclerotic lesions, leading to an increased risk of myocardial infarction or stroke. Here, platelet activation plays a major role, leading to the release of bioactive molecules, for example, chemokines and coagulation factors, and to platelet clot formation. Several antiplatelet therapies have been developed for secondary prevention of cardiovascular events, in which anticoagulant drugs are often combined. Besides playing a role in hemostasis, platelets are also involved in inflammation. However, it is unclear whether current antiplatelet therapies also affect platelet immune functions. In this study, the possible anti-inflammatory effects of antiplatelet medications on chemokine release were investigated using enzyme-linked immunosorbent assay and on the chemotaxis of THP-1 cells toward platelet releasates. We found that antiplatelet medication acetylsalicylic acid (ASA) led to reduced chemokine (CC motif) ligand 5 (CCL5) and chemokine (CXC motif) ligand 4 (CXCL4) release from platelets, while leukocyte chemotaxis was not affected. Depending on the agonist, α _IIb_ β _3_ and P2Y _12_ inhibitors also affected CCL5 or CXCL4 release. The combination of ASA with a P2Y _12_ inhibitor or a phosphodiesterase (PDE) inhibitor did not lead to an additive reduction in CCL5 or CXCL4 release. Interestingly, these combinations did reduce leukocyte chemotaxis. This study provides evidence that combined therapy of ASA and a P2Y _12_ or PDE3 inhibitor can decrease the inflammatory leukocyte recruiting potential of the releasate of activated platelets.

## Introduction

Atherothrombosis, a result of atherosclerotic plaque rupture or erosion, can lead to acute coronary syndrome (ACS), ischemic strokes, and cardiovascular deaths and contributes to the global burden of premature mortality and morbidity. 1 Platelet activation plays a central role in atherothrombosis, which in turn leads to the release of prothrombotic and proinflammatory factors and amplifies activation of the coagulation cascade. 2 3 Although the importance of platelets in the acute phase of cardiovascular disease (CVD) is undisputed, their relevance for the development of atherosclerosis is incompletely understood. Many studies have highlighted functions of platelets beyond hemostasis. 4 For example, platelets can bridge leukocytes to the inflamed vessel wall, 5 6 7 they release extracellular vesicles with proinflammatory activity, 8 9 and they can induce the release of neutrophil extracellular traps. 10 11 In addition, platelets also release chemokines from α-granules upon activation. 12 13

Chemokines are a group of small chemotactic cytokines that orchestrate cell trafficking and play important roles in immune responses, inflammation, angiogenesis, and cell differentiation. 14 The CC and CXC chemokines are the largest subfamilies. The CXC-chemokine ligand 4, CXCL4 (platelet factor 4), is almost exclusively expressed in platelets and the fourth most abundant platelet protein (355,000 copies per platelet). 15 Proteomic analysis suggests that CC-chemokine ligand 5, CCL5 (RANTES), is the only CC-chemokine expressed in relevant amounts in platelets (approximately 4,500 copies per platelet). 15 Platelet activation leads to CCL5 and CXCL4 release from the α-granules and both chemokines can also be deposited on inflamed endothelium and lead to subsequent monocyte arrest. 16 In addition, binding of CCL5 to CXCL4 increases monocyte arrest to endothelial cells under flow. 17 18 Besides facilitating CCL5-induced monocyte arrest, CXCL4 has several reported physiologic functions, for example, modifying differentiation of T-cells and macrophages, activation of smooth muscle cells, inhibition of apoptosis of neutrophils and monocytes, and increasing oxidized low-density lipoprotein uptake. 19

Control of platelet reactivity is essential for the secondary prevention of adverse cardiovascular events. 20 21 After myocardial infarction, “dual antiplatelet therapy,” that is, combined treatment with the cyclooxygenase (COX) inhibitor acetylsalicylic acid (ASA; aspirin) and purinergic receptor P2Y _12_ antagonists, for example, clopidogrel, prasugrel, or ticagrelor, is recommended. For immediate platelet effects, the intravenous P2Y _12_ antagonist cangrelor or ɑ _IIb_ β _3_ antagonists are available. Finally, cilostazol is a phosphodiesterase 3 (PDE3) inhibitor and is implemented as a treatment for patients with peripheral arterial disease. 22 Of note, most platelet inhibition strategies bear a nonnegligible risk of severe bleeding complications. In addition, a substantial number of patients does not optimally respond to antiplatelet therapy. 23

During antiplatelet therapy, a reduction in inflammation was observed in patients. 24 However, it is unclear whether this is due to direct effects of antiplatelet therapy on platelets or indirect, nonplatelet-dependent effects. 24 The aim of this study is to investigate the influence of common antiplatelet drugs on inflammatory functions of platelets and whether this influence is distinct from their established antihemostatic effects. Serving as a model for the inflammatory function of platelets, the release of chemokines by platelets from healthy donors and the chemotactic properties of platelets toward monocytic THP-1 cells were determined, after treatment with antiplatelet drugs. This study provides additional evidence that the anti-inflammatory effects seen in clinical trials might originate from platelets, depending on the pathway of platelet activation.

## Materials and Methods

Evasin-4 was expressed in *Escherichia coli* , purified by high-performance liquid chromatography and refolded as described. 25 All other reagents were at the highest purity available and obtained from Merck (Darmstadt, Germany), unless indicated.

### Platelet Isolation and Activation

Blood was collected from healthy volunteers and two patients with Glanzmann thrombasthenia, with established deficiency in integrin α _IIb_ β _3_ , 26 with a 21 Gauge needle (vacutainer precision glide, BD) into citrate tubes (9 mL coagulation sodium citrate 3.2% vacuette, Greiner Bio-One, Kremsmünster, Austria). For the condition in the presence of aspirin, donors were given aspirin orally (100 mg Bayer, Leverkusen, Germany) the evening before blood donation. Platelet-rich plasma (PRP) was obtained by centrifugation of blood at 350 g for 15 minutes. Washed platelets were obtained by centrifugation of PRP at 1,240 g for 15 minutes, and a wash step with platelet buffer pH 6.6 (10 mM 4-(2-hydroxyethyl)-1-piperazineethanesulfonic acid [HEPES] buffer, 2 mM CaCl _2_ , 136 mM NaCl, 2.7 mM KCl, and 2 mM MgCl _2_ supplemented with 0.5% bovine serum albumin [BSA] and 0.2% glucose). All centrifugation steps were performed in presence of anticoagulant acid citrate buffer (80 mM trisodium citrate, 52 mM citric acid, and 183 mM glucose), to prevent platelet activation during isolation procedure. After pelleting, platelets were resuspended in platelet buffer pH 7.45 (10 mM HEPES buffer, 2 mM CaCl _2_ , 136 mM NaCl, 2.7 mM KCl, and 2 mM MgCl _2_ supplemented with 0.5% BSA and 0.2% glucose) at a concentration of 2 × 10 ^8^ platelets/mL. The inclusion of human subjects was approved after full informed consent by the local Maastricht ethics committee, and studies were performed in accordance with the declaration of Helsinki.

Washed platelets (2 × 10 ^8^ /mL) were activated with different agonists, 100 ng/mL convulxin (CVX, Enzo Life Sciences, Lausen, Switzerland), 50 µM TRAP-6 (AnaSpec Inc. California, United States), or 5 nM thrombin (Haematologic Technologies, New Hampshire, United States) for 30 minutes at 37°C. Platelets were preincubated for 5 minutes at 37°C with inhibitors prior to activation, except cilostazol (10 minutes). Integrin α _IIb_ β _3_ ligand binding was blocked with 10 µM tirofiban (CAS 144494–65–5, Correvio Int., Geneva, Switzerland) or 10 µM eptifibatide (Integrilin, CAS 188627–80–7, GlaxoSmithKline, Brentford, United Kingdom). P2Y _12_ was inhibited with 1 µM cangrelor (CAS 163706–06–7, Novartis, Basel, Switzerland), PDE3 with 5 µM cilostazol (CAS 73963–72–1, Tebu Bio, Le Perray-en-Yvelines, France), and inhibition of thromboxane A2 (TXA2) generation with 100 mg aspirin (ASA, CAS 50–78–2, Bayer, Leverkusen, Germany) ingested by donors the evening before blood donation. Activated platelets were spun down by centrifugation at 300 g for 5 minutes, after which the supernatant was filtered with PK50 MiniSart sterile 0.8 µm filters (Sartorius, Göttingen, Germany) and centrifugated for 1 hour at 20,000 g. Samples were collected and snap frozen into liquid nitrogen and stored at –80°C until analyses.

### Chemokine and Serotonin Determination

Washed platelets (2 × 10 ^8^ /mL) were activated as described and after time points (5, 15, 30, and 60 minutes) chemokine samples were collected. Secretion of chemokine CCL5 was determined by an in-house enzyme-linked immunosorbent assay (ELISA); CXCL4 and serotonin secretion were determined by an ELISA kit from R&D Systems (Minneapolis, Minnesota, United States) and Abnova (Taipei, Taiwan) according to manufacturer's instructions, respectively. For CCL5, samples were diluted into phosphate buffered saline (PBS) with 1% BSA, and incubated for 2 hours at room temperature in a Maxisorb 96-well plate (Nunc), coated with CCL5 capture antibody (R&D Systems, Minnesota, United States). After washing with PBS buffer containing 0.05% Tween-20, a second antibody (biotin-labeled goat antihuman CCL5 mAb, home-made) was added, and incubated for 2 hours at room temperature. For detection, incubation with HRP-labeled streptavidin (R&D Systems) was performed in the dark for 20 minutes at room temperature. A TMB substrate kit (KPL Inc., Massachusetts, United States) was used and color development was measured at 450 nm and 550 nm wavelengths. Data analysis was performed with a four-parameter logistic fit calculation.

### Cell Migration Assay

For assessment of THP-1 cell migration toward a chemoattractant, a 12-well Boyden chemotaxis chamber (NeuroProbe, Gaithersburg, Germany) with a 5 µm pore polycarbonate membrane (NeuroProbe, Gaithersburg, Germany) was used. The chemoattractants are the supernatants after platelet activation. Donor samples were pooled per condition and diluted four times in RPMI 1% FBS medium (Thermo Fisher Scientific, Massachusetts, United States). Chemoattractants were added to the lower compartment of the chamber. In some experiments, the tick-derived CC-chemokine inhibitor Evasin-4 was added at 1 µg/mL. THP-1 cells in a concentration of 10 ^6^ /mL cells were added to the upper compartment of the chamber. After incubation of 1.5 hours at 37°C, the membrane was cleared of nonmigrated cells and the membrane was stained with Diff-Quick stain (Eberhard Lehmann GmbH, Berlin, Germany). Stained membrane was imaged with light microscopy (Leica), and cells were counted manually in five fields per well and expressed as cells/mm ^2^ . The migration assay was done at least four times per condition.

### Statistical Analysis

Independent and unpaired experiments were performed using platelets from a total of 38 different healthy blood donors to investigate the effects of antiplatelet drugs. The donor platelets were used for the (buffer) controls and for the treatment with the different compounds. Control groups contained all untreated platelets and were thus higher in number than the treatment groups. Experimental data were represented as median with interquartile range or as mean ± standard deviation. Statistical analysis was performed with one-way analysis of variance or with Kruskal–Wallis test with Sidak or Dunn's post hoc analysis, where applicable. Significance of differences of a *p* -value <0.05 were considered significant. Statistical analysis was performed with Graphpad Prism software version 9.2.

## Results

### Release of Chemokines from Activated Platelets Is Not Dependent on Activation Pathway

**Fig. 1:**
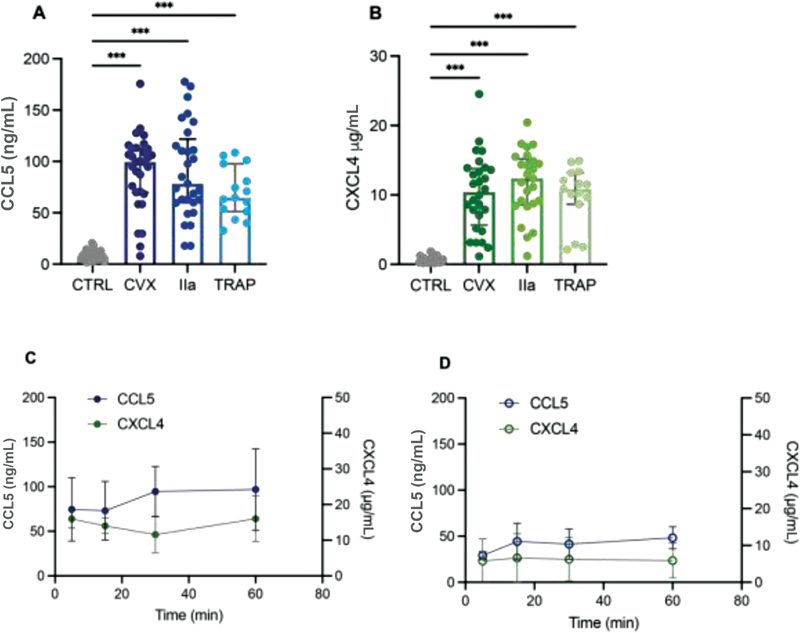
Different platelet activation pathways have no influence on chemokine release. Washed platelets (2 × 10 ^8^ /mL) were activated with convulxin (CVX, 100 ng/mL), thrombin (IIa, 5 nM), or TRAP-6 (50 µM) for 30 minutes at 37°C. Platelets were removed and chemokines CCL5 ( **A** ) and CXCL4 ( **B** ) were determined with enzyme-linked immune sorbent assay. Control (CTRL) represents no stimulated platelets. Chemokine release was followed in time after convulxin ( **C** ) and thrombin ( **D** ) activation. Closed circles represent convulxin activation and open circles represent thrombin activation. CTRL: *n* = 31; CVX: *n* = 28–30, IIa: *n* = 25–29; and TRAP-6: *n* = 15. Median with interquartile range ( **A, B** ); mean ± standard deviation ( **C, D** ). *** *p* < 0.001, Kruskal–Wallis with Dunn's test.

Platelet activation leads to release of their content, for example, coagulation and growth factors, chemokines, and of extracellular vesicles. In this study, a focus lies on the release of the chemokines CXCL4 and CCL5. Platelet activation by convulxin (glycoprotein VI [GPVI] agonist), thrombin (protease-activated receptor [PAR]1/PAR4 agonist), and TRAP-6 (PAR1 agonist) led to comparable levels of released chemokine ( Fig. 1A , B ). Intriguingly, there was a notable donor-to-donor difference regarding chemokine release by activated platelets ( Fig. 1A , B ). Already after 5 minutes of platelet activation, maximum levels of CCL5 and CXCL4 were observed with both convulxin and thrombin stimulations ( Fig. 1C , D ). These findings indicate that activated platelets release chemokines rapidly upon stimulation of GPVI or PAR1/PAR4 receptors.

### Impact of Platelet Aggregation Inhibitors on CCL5 and CXCL4 Release by Platelets

**Fig. 2:**
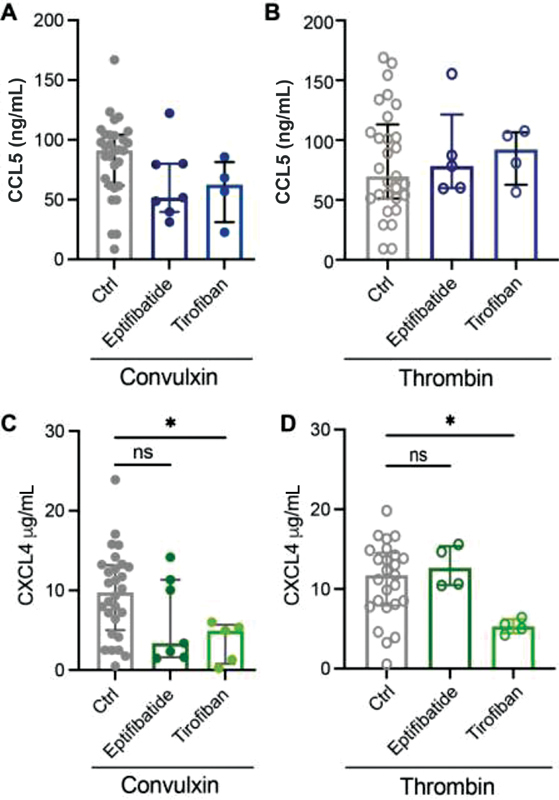
Effects of antiplatelet drugs against α _IIb_ β _3_ on chemokine release. Washed platelets (2 × 10 ^8^ /mL) were incubated with indicated compounds, 5 minutes prior to platelet activation, and chemokine release was determined: CCL5 release after convulxin ( **A** ) and thrombin ( **B** ) activation and CXCL4 release after convulxin ( **C** ) and thrombin ( **D** ) activation. Closed circles represent convulxin activation and open circles represent thrombin activation. CTRL: *n* = 25–30; eptifibatide: *n* = 4–7; and tirofiban: *n* = 4–5. Median with interquartile range. * *p* < 0.05, Kruskal–Wallis with Dunn's test.

Some clinical studies suggested that inhibition of α _IIb_ β _3_ integrin, responsible for platelet aggregation, reduces the inflammatory response in patients. 24 To investigate whether platelet aggregation inhibitors can also inhibit chemokine release, washed platelets were incubated with eptifibatide or tirofiban for 5 minutes prior to platelet activation with convulxin or thrombin. The release of CCL5 was not significantly reduced after antiplatelet treatment ( Fig. 2A , B ). Interestingly, whereas eptifibatide hardly showed an effect, the release of chemokine CXCL4 was decreased by over 50% after treatment with tirofiban ( Fig. 2C , D ). This difference in CCL5 and CXCL4 release was also observed in platelets isolated from two patients with Glanzmann thrombasthenia, who have defective α _IIb_ β _3_ integrins ( Supplementary Figure S1 ). These data suggest that the chemokines CCL5 and CXCL4 are released by differential pathways. Taken together, these findings imply that inhibition of integrin α _IIb_ β _3_ only has minor effects on chemokine release from activated platelets.

### Single or Dual Antiplatelet Therapy Influences CCL5 and CXCL4 Release

**Fig. 3:**
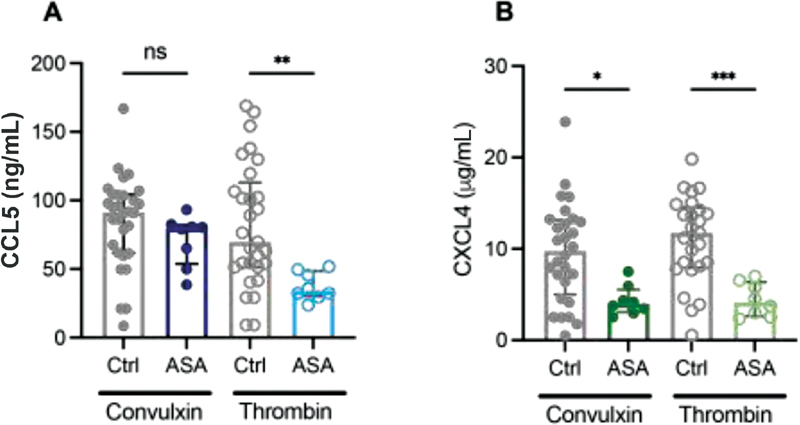
Effect of acetylsalicylic acid (ASA) on chemokine release. Washed platelets (2 × 10 ^8^ /mL) from healthy volunteers exposed to ASA (100 mg p.o.) were activated and CCL5 ( **A** ) and CXCL4 ( **B** ) release was determined as described. Closed circles represent convulxin activation and open circles represent thrombin activation. CTRL: *n* = 25–30; and ASA: *n* = 8. Median with interquartile range. * *p* < 0.05; ** *p* < 0.01; *** *p* < 0.001; Kruskal-Wallis with Dunn's test.

**Fig. 4:**
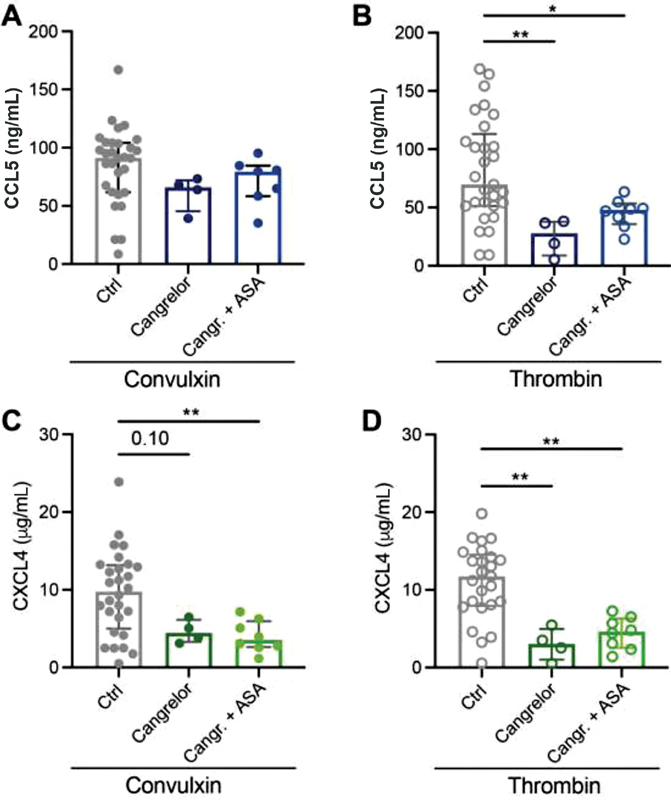
Effects of P2Y _12_ inhibition on chemokine release. Washed platelets (2 × 10 ^8^ /mL) from healthy volunteers exposed to acetylsalicylic acid (ASA: 100 mg p.o.) were activated and CCL5 ( **A, B** ) and CXCL4 ( **C, D** ) release was determined as described. Prior to activation, platelets were incubated with P2Y _12_ inhibitor cangrelor for 5 minutes. Closed circles represent convulxin activation and open circles represent thrombin activation. CTRL: *n* = 25–30; Cangrelor: *n* = 4; Cangr. + ASA: *n* = 8. Median with interquartile range. * *p* < 0.05; ** *p* < 0.01; Kruskal–Wallis with Dunn's test.

ASA and P2Y _12_ inhibitors are commonly prescribed antiplatelet drugs for the secondary prevention of major adverse cardiovascular events. 27 28 Platelet inhibition with ASA did not show a significant effect on CCL5 release from convulxin-activated platelets, whereas CCL5 release after thrombin activation was reduced ( Fig. 3A ). Interestingly, unlike CCL5, CXCL4 chemokine was reduced after stimulation of convulxin or thrombin ( Fig. 3B ). Similar to ASA, the release of CCL5 was not affected by cangrelor after stimulation of the GPVI pathway using convulxin ( Fig. 4A ). However, CCL5 release was reduced by cangrelor after stimulation of the PAR1/PAR4 pathway with thrombin ( Fig. 4B ). The release of CXCL4 was reduced by cangrelor after stimulation with thrombin and a downward trend ( *p* = 0.1) was observed upon stimulation with convulxin ( Fig. 4C , D ). Combined treatment of platelets with both ASA and cangrelor did not further increase the overall inhibition of chemokine release ( Fig. 4A – D ).

### Impact of Combined Cilostazol and ASA Treatment on CCL5 and CXCL4 Release from Activated Platelets

**Fig. 5:**
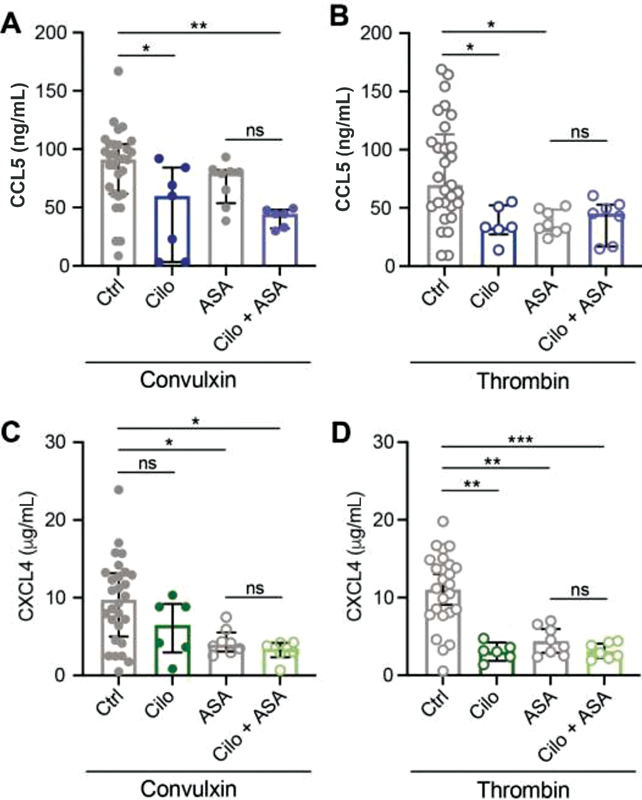
Dual treatment with acetylsalicylic acid (ASA) + cilostazol treatment has no additional effects on chemokine release. Washed platelets (2 × 10 ^8^ /mL) from healthy volunteers exposed to ASA (100 mg p.o.) were activated and CCL5 ( **A, B** ) and CXCL4 ( **C, D** ) release was determined as described. Platelets were incubated 10 minutes prior to activation with the PDE3 inhibitor cilostazol. Closed circles represent convulxin activation and open circles represent thrombin activation. CTRL: *n* = 25–29; Cilo: *n* = 6; ASA: *n* = 8; and ASA + Cilo: *n* = 6. Median with interquartile range. * *p* < 0.05; ** *p* < 0.01; and *** *p* < 0.001; analysis of variance with Sidak test.

**Fig. 6:**
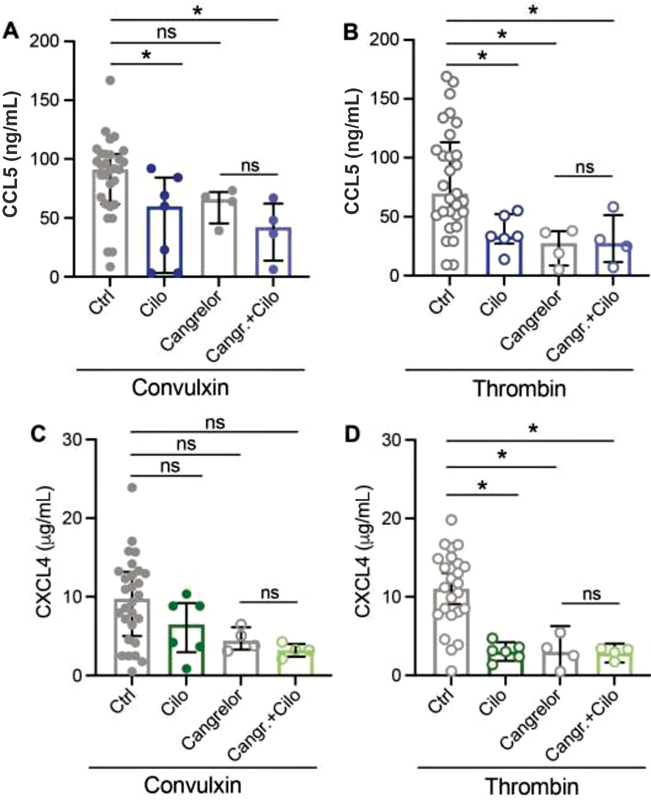
Combination treatment with cangrelor + cilostazol has no additional effects on chemokine release. Washed platelets (2 × 10 ^8^ /mL) from healthy volunteers were pretreated with P2Y _12_ inhibitor cangrelor, 5 minutes before activation and CCL5 ( **A, B** ) and CXCL4 ( **C, D** ) release was determined as described. Platelets were incubated 10 minutes prior to activation with the PDE3 inhibitor cilostazol. Closed circles represent convulxin activation and open circles represent thrombin activation. CTRL: *n* = 25–30; Cilo: *n* = 6–7; Cangrelor: *n* = 4; and Cangr. + Cilo: *n* = 4. Median with interquartile range. * *p* < 0.05 and ** *p* < 0.01, analysis of variance with Sidak test.

In accordance with our recent observations, 29 inhibition of platelet cAMP via PDE3 with cilostazol was shown to have an inhibiting effect on chemokine CCL5 release upon stimulation with both convulxin and thrombin ( Figs. 5A , B and 6A , B ), while CXCL4 release was significantly inhibited by cilostazol only after stimulation with thrombin ( Figs. 5C , D and 6C , D ). To investigate whether cilostazol has an additional effect on CCL5 and CXCL4 release from ASA-treated platelets, these platelets were incubated with cilostazol for 10 minutes prior to platelet activation. This only resulted in a minimal decrease in chemokine release compared with ASA alone ( Fig. 5 ), except when CCL5 release was measured after triggering with convulxin ( Fig. 5A ). Here addition of cilostazol resulted in a stronger decrease in CCL5 release than ASA alone ( Fig. 5A ), but this effect was not statistically significant. The combination of cilostazol with cangrelor had no additional effect on the release of CCL5 and CXCL4 ( Fig. 6 ). These data suggest that combined treatment of platelets with ASA and cilostazol does not potentiate the inhibition of chemokine release after platelet activation.

Platelet-derived serotonin was found to mediate proinflammatory roles during myocardial infarction and during systemic shock. 30 31 To investigate the effects of antiplatelet drugs on the release of serotonin from platelets after activation with convulxin or thrombin, serotonin was determined in platelet releasates after treatment. Interestingly, only the presence of cangrelor inhibited serotonin release induced by either agonist ( Supplementary Figure S2 ).

### Combined Treatment of Platelets with Aspirin and Cangrelor or Cilostazol Inhibits Chemotaxis of Monocytic Cells

**Fig. 7:**
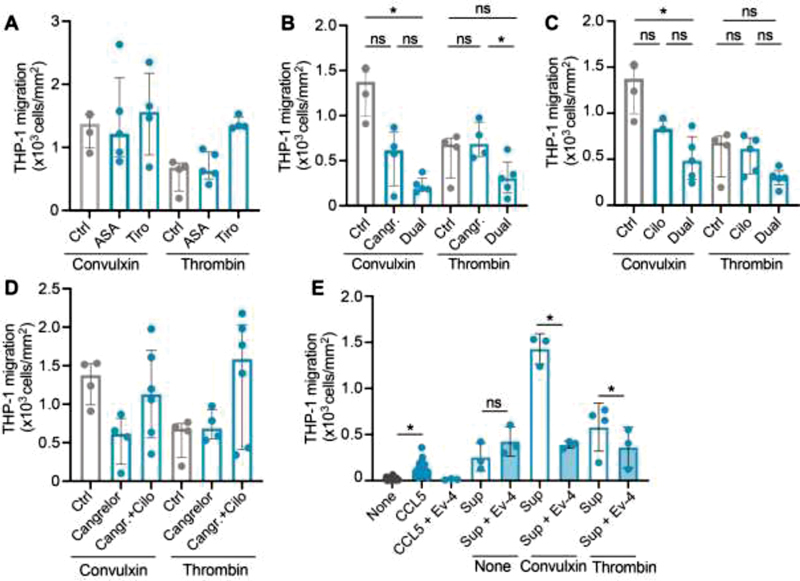
Chemoattractant properties of activated platelets and the effects of antiplatelet drugs. Migration of monocytic cells (10 ^6^ /mL) was induced in a 12-well chemotaxis chamber for 90 minutes at 37°C. Buffer, or supernatants of resting or activated washed platelets, was added in the bottom compartment. If applicable, platelets were activated by convulxin or thrombin without or with acetylsalicylic acid (ASA) or tirofiban ( **A** ), cangrelor or ASA + cangrelor (dual) ( **B** ), cilostazol (cilo) or ASA + cilo (dual) ( **C** ) **,** and cangrelor or cangrelor + cilo ( **D** ). CCL5 (0.5 µg/mL) and platelet releasates without or with Evasin-4 (Ev-4, 1 µg/mL) ( **E** ). *n* = 4–6; median with interquartile range. * *p* < 0.05; Kruskal–Wallis with Dunn's test.

Chemokines CCL5 and CXCL4 are involved in various immune pathways, for example migration and adhesion of leukocytes. To investigate possible effects of antiplatelet drugs on platelet-induced leukocyte migration, a Boyden chemotaxis chamber was used to assess migration of monocytic THP-1 cells toward platelet supernatants. Releasates of platelets activated with convulxin induced a more pronounced chemotactic response than those induced after activation with thrombin ( Fig. 7 ). Platelet activation after exposure to ASA or tirofiban did not lead to a reduced migration with both agonists ( Fig. 7A ). Interestingly, the chemotactic potential of platelets releasate was reduced after inhibition with cangrelor, but only when activated with convulxin ( Fig. 7B ). This inhibition was more pronounced when cangrelor was combined with ASA ( Fig. 7B ). Inhibition of platelets with cilostazol alone led to a slight decrease in migration, which could be further reduced by a combination with ASA ( Fig. 7C ). The combination of cangrelor and cilostazol had no effect of monocytic cell migration ( Fig. 7D ). Furthermore, the inhibitors themselves have no influence on migration of monocytic cells, both in the absence and presence of CCL5 as chemoattractant ( Supplementary Figure S3 ).

To further investigate whether the reduction in chemotaxis could be due to a reduced release of CCL5, chemotaxis experiments were performed in the presence of the broad-spectrum CC-chemokine tick-derived antagonist Evasin-4. Indeed, Evasin-4 abolished chemotaxis toward CCL5, and THP-1 migration toward the supernatants of both convulxin- and thrombin-activated platelets was strongly reduced in the presence of Evasin-4 ( Fig. 7E ).

Taken together, these data suggest that combined therapy of ASA and P2Y _12_ or PDE3 inhibitors can decrease the inflammatory leukocyte recruiting potential of the releasate of activated platelets, possibly by inhibiting the release of CCL5.

## Discussion

In this study, we investigated the effect of antiplatelet medication on platelet-chemokine release and platelet releasate-induced chemotaxis. We focused on convulxin and thrombin as these agonists potently trigger protein kinase C activation, which is critical for platelet granule secretion 32 as chemokines CCL5 and CXCL4 reside in α-granules. 16 We could confirm previous observations that chemokine release is a rapid response after platelet activation and occurs nearly instantaneously within 5 minutes after activation. 16 33 Interestingly, although activation with convulxin and thrombin led to similar amounts of CCL5 and CXCL4 released along with a similar time course, release of CCL5 induced by convulxin alone appeared to be more resistant to antiplatelet compounds than the release of CXCL4. A clear reduction in CXCL4 release was observed after treatment of platelets with tirofiban. This effect was less pronounced when eptifibatide was used. Interestingly, there was no reduction in CCL5 release after incubation with any ɑ _IIb_ β _3_ inhibitor, neither was CCL5 or CXCL4 release reduced in platelets from the two Glanzmann patients. However, tirofiban appeared to not interfere with other chemoattractants released by platelets, as it did not influence migration of monocytic cells. It should be taken into account that ɑ _IIb_ β _3_ antagonists on the market are both structurally and functionally different, which leads to different outcomes in different studies. For example, abciximab is a humanized fab fragment of the monoclonal 7e3, the cyclic peptide eptifibatide is not only specific to α _IIb_ β _3_ integrin but also binds to α _M_ β _2_ and to ɑ _v_ β _3_ , and tirofiban is considered to be specific for α _IIb_ β _3_ integrin and binds to the RGD binding site on the integrin, which might lead to neoepitopes. 24 34 35 In animal models, blockade or genetic deletion of α _IIb_ β _3_ reduced platelet interactions with the endothelium and with leukocytes. 36 37 This was also observed in models with human platelets and endothelial cells 38 and in patients with ACS. 39 40 In our study, we investigated platelet releasate-induced leukocyte migration but did not study direct interaction of platelet (-chemokines) with leukocytes and/or the endothelium. Regarding the findings in this study, it can be stated that depending on which platelet-derived chemokines are investigated, there is an anti-inflammatory effect of these drugs.

ASA is well known for its antiplatelet and anti-inflammatory effects. ASA irreversibly acetylates COX-1 and COX-2, thereby inhibiting the production of TXA2 via COX-1, leading to inhibition of platelet aggregation and decreased vasoconstriction. 41 A low dose (81–100 mg 24 ) of ASA has anti-inflammatory effects, by triggering the synthesis of arachidonic acid metabolites leading to blockade of the expression of CXCL8 in macrophages and endothelial cells. 24 42 In this study, we have observed that ASA significantly decreased chemokine release through the thrombin-induced pathway (PAR1/PAR4), and to a lesser extent after activation with convulxin. Despite the observed reduction in chemokine release, the anti-inflammatory response of ASA was not reflected in the migration of monocytes in this study, which was unaffected by ASA. This may suggest that ASA mediates its anti-inflammatory response mainly in a platelet-independent manner.

A resistance of patients toward ASA leads to suboptimal antiplatelet therapy. 23 This issue is addressed, for example, by combining ASA with a second antiplatelet drug, for example, P2Y _12_ receptor inhibitors (clopidogrel, ticagrelor, prasugrel). Unlike for clopidogrel, ticagrelor and cangrelor have less data available on their influence on circulating markers of inflammation in patients, although ticagrelor more efficiently reduced CXCL8 levels in healthy volunteers than clopidogrel. 43 Clopidogrel was shown to reduce inflammatory markers in CVD patients, and it can interfere with leukocyte–platelet interactions, although it is unclear whether this is due to vascular or antiplatelet effects. 43 44 45 46 Furthermore, clopidogrel reduced CCL5 plasma levels both in animals and in patients. 47 48 49 All P2Y _12_ antagonists appear to interfere with the interaction of platelets with monocytes and with neutrophils, 43 45 50 although all may have platelet-independent effects, as stated above. We have observed that treatment of platelets with cangrelor showed a similar effect as with ASA. The chemokine release induced by thrombin is inhibited by cangrelor, whereas chemokine release induced by convulxin was less well inhibited by cangrelor. Interestingly, cangrelor was the only compound that blocked the release of serotonin. Inhibition of platelets with cangrelor alone did not lead to a reduced migration of monocytic cells. Although cangrelor in combination with ASA did not lead to a further reduction in chemokine release compared with ASA or cangrelor alone, combination of both compounds almost eliminated attraction of monocytic cells by platelet supernatant. A possible explanation for this observation might be that the combination of ASA and cangrelor can inhibit the release of several chemoattractants from platelets, other than CCL5. One possible chemoattractant is adenosine diphosphate (ADP), released from dense granules by activated platelets, and was shown to attract monocytes and macrophages through the action of the P2Y _12_ receptor in recent studies. 50 51 52 Although our results indicated that CCL5 was mainly responsible for the chemotactic effect of platelet supernatants, an involvement of ADP appears feasible since remnant levels of cangrelor in the platelet supernatants might be sufficient to reduce chemotaxis. In addition, cangrelor inhibited the release of serotonin, which is likewise stored in dense granules, and this could explain why a strong inhibition of monocyte chemotaxis toward supernatants of cangrelor + ASA-treated platelets was observed, while CCL5 secretion was poorly affected by this combined treatment. Thus, besides chemokines, antiplatelet drugs can also affect the release of other compounds that mediate monocyte and macrophage migration. For future studies, it would be interesting to compare the chemotactic effects of platelet supernatants treated without or with apyrase, an enzyme that hydrolyzes ADP. Outside the context of platelets and their supernatants, no direct effects of the antiplatelet drugs cangrelor, tirofiban, and cilostazol were found on the chemotaxis of THP-1 cells, both in the presence and absence of CCL5, indicating that the observed effects in this study can be attributed to the actions of these drugs on platelets.

So far, this study has focused on the effect of antiplatelet medications and combinations on the inflammatory properties of platelets. Interestingly, we have observed differential protein and extracellular vesicle secretion patterns after platelet activation throughout this, and in other studies. 19 29 53 54 In this study, we have observed differential CCL5 and CXCL4 release under the influence of different antiplatelet medications. This would suggest that these chemokines are differently packaged inside the α-granule of platelets and that their differential release is governed by autocrine feedback activation mechanisms. Although differential packaging and release of granule content has been described previously in literature, it remains controversial whether this is a physiologic regulatory principle 55 56 or a stochastically occurring process. 57 58 Support for the latter comes from studies that show that platelet secretion depends on several factors, for example, cargo solubility, granule shape, and/or granule–plasma membrane fusion routes. 57 In addition, α-granule proteins were found to be stochastically stored in the granules into subdomains. 58 Others did find evidence for a functional separation of α-granule content and of their release depending on the context of platelet activation. 55 56 Unlike the previous studies, this study also took the effects of inhibitors of platelet activation and activation into account, thereby revealing a differential release of α-granule content.

PDE3 and PDE5 regulate the cAMP- and cGMP-dependent signaling pathways in platelets, and the PDE3 inhibitor cilostazol was shown to inhibit platelet aggregation and the release of P-selectin, CXCL4, and platelet-derived growth factor in previous studies 29 (reviewed in 59 ). Moreover, inhibition of PDE3 by cilostazol also decreased monocyte recruitment. 29 In this study, the combination of ASA and cilostazol did not further inhibit chemokine release after platelet activation compared with ASA alone. However, when combined, monocyte recruitment was decreased, which suggests that the combination of ASA and cilostazol can inhibit the release of chemoattractants from platelets. Interestingly, the addition of the CC-chemokine inhibitor Evasin-4 led to a strong reduction in THP-1 chemotaxis toward both convulxin- and thrombin-induced platelet releasates. Although Evasin-4 blocks many CC-chemokines, a proteomics study only detected CCL5 as a CC-chemokine member within platelets. 15 In addition, CXCL4, which is unaffected by Evasin-4, poorly affects monocyte recruitment. 17 18 This indicates that, at least in this experimental setting, CCL5 is mainly responsible for the chemotactic effects of platelet releasates.

In summary, on basis of our findings we can conclude that the majority of antiplatelet drugs influence the release of inflammatory mediators, chemokines in this study, from activated platelets. Although ASA, P2Y _12_ receptor inhibitors, and PDE3 inhibitors also have an effect on the vasculature and leukocytes, they are also able to reduce inflammation in a platelet-dependent manner, for example, by modulating interactions of platelets with other immune cells, 43 45 and by inhibition of platelet secretion through the thrombin activation pathway (this study). Interestingly, chemokine release from platelets can be effectively reduced by specific combinations of medications. Dual therapy with ASA and a P2Y _12_ receptor inhibitor or with cilostazol shows promising effects in reducing the proinflammatory properties of platelets. Whether antiplatelet drugs can be used to reduce low-grade inflammation, a possible driver of CVD, 60 remains to be determined and it is challenging to pinpoint such effects on platelets. In addition, given the beneficial effects of platelets and their released contents in wound-healing processes, 61 inhibition of chemokine release might not always be advantageous.

Nevertheless, for patients with CVD and notably with atherothrombosis, the reduction in inflammation by targeting of chemokine release during antiplatelet treatment could be supplemented with an anticoagulant, for example, rivaroxaban (direct antifactor Xa inhibitor), to further prevent disease progression and manifestation while minimizing the risk for bleeding complications.
