# Supplementary material for: Combined Antiplatelet Therapy Reduces the Proinflammatory Properties of Activated Platelets
Source: TH Open. 2021 Oct 28;5(4):e533–42. doi: 10.1055/a-1682-3415 (PMC8651446; doi:10.1055/a-1682-3415)
Supplement: Supplementary file 1 — Supplementary Material [file 10-1055-a-1682-3415-s210047.pdf]

Combined antiplatelet therapy reduces the pro-inflammatory properties of activated platelets

Alexandra CA Heinzmann, Daniëlle M Coenen, Tanja Vajen, Judith MEM Cosemans, Rory R Koenen

Supplementary figures

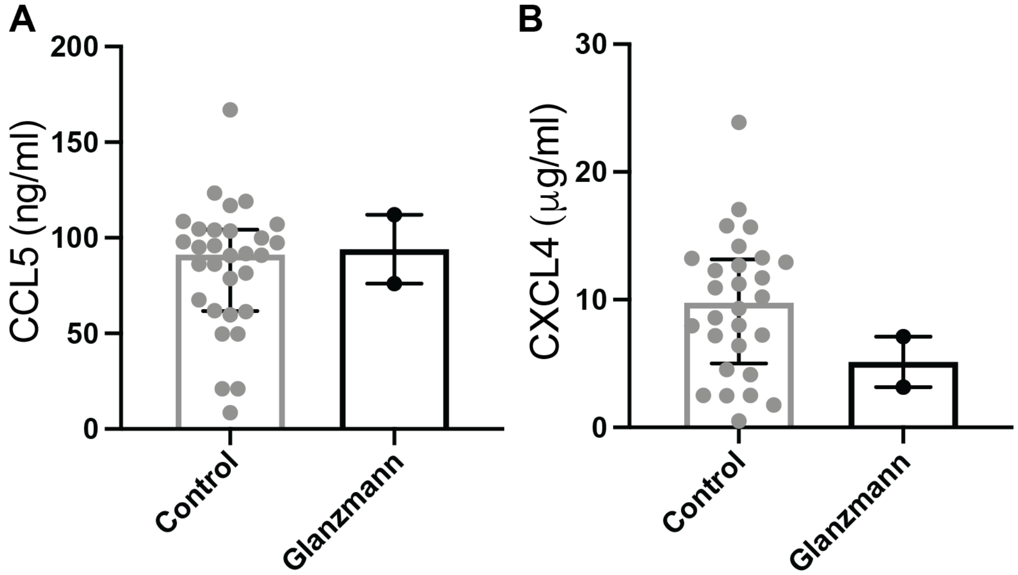

Figure S1: Effects of $\alpha_{IIb}\beta_3$-deficiency on chemokine release. Washed platelets (2x10^8/ml) isolated from individuals with Glanzmann thrombasthenia were activated with convulxin **(A,B)** and chemokines were determined as described. Control: n=28-30, Glanzmann: n=2, median ± interquartile range.

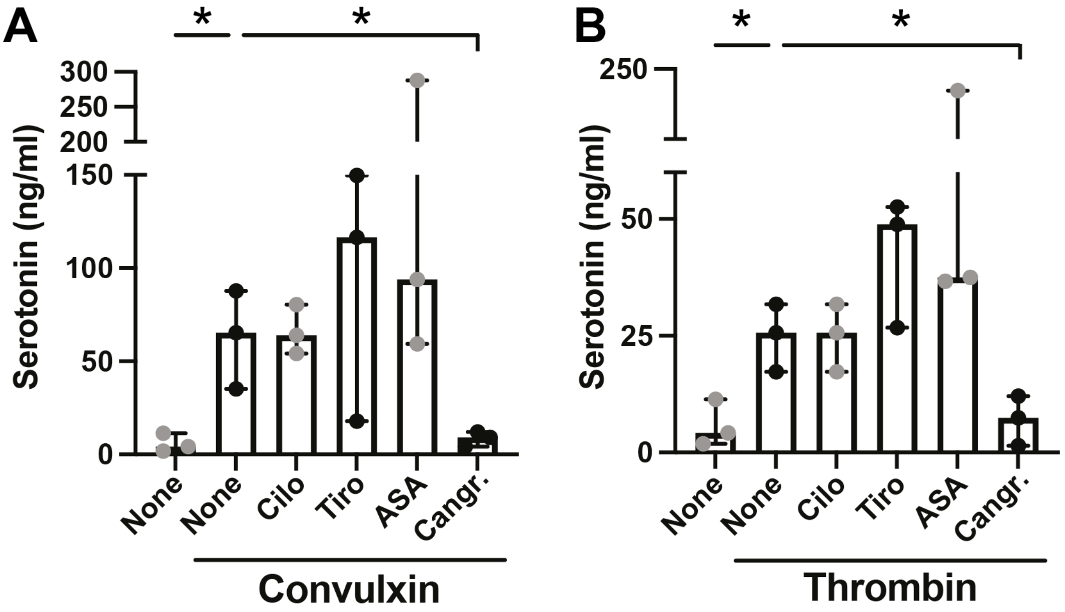

Figure S2: Effect of antiplatelet drugs on serotonin release. Washed platelets (2x10^8/mL) from healthy volunteers exposed to ASA (100 mg p.o.), or washed platelets treated with cilostazol (5 μM), tirofiban (10 μM) and cangrelor (1 μM) for 10 min at 37°C, prior to activation with convulxin (100 ng/ml) **(A)** or thrombin (5 nM) **(B)** and serotonin release was determined as described. n=3, median ± interquartile range. *P< 0.05. Kruskal-Wallis with Dunn's test.

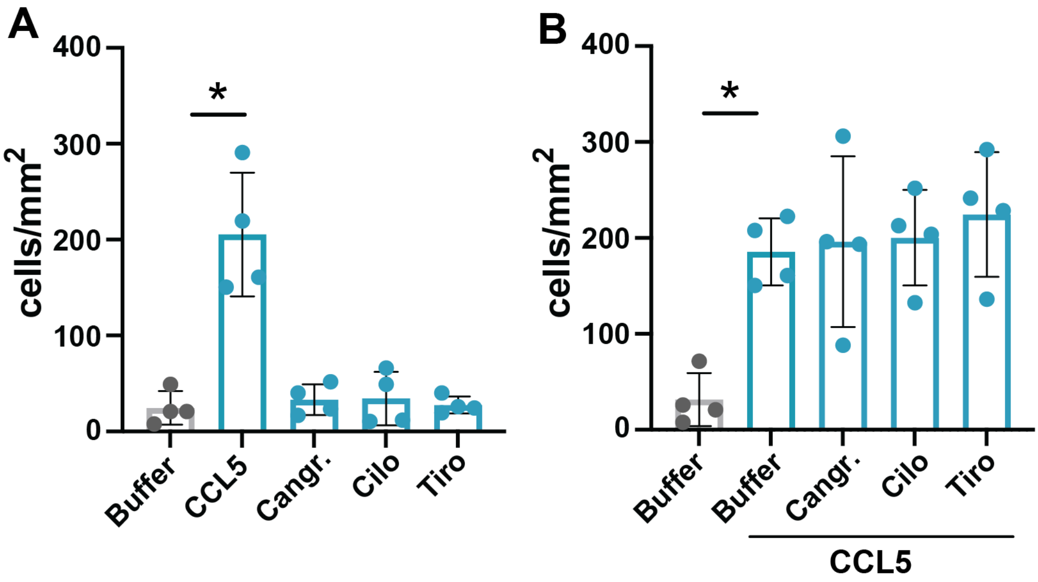

Figure S3: The effects of antiplatelet drugs on the chemotaxis of THP-1 cells. Migration of monocytic cells (1 x 10^6/mL) was induced in a 12-well chemotaxis chamber for 90 min at 37°C. Buffer or CCL5 (0.5µg/mL) was added in the bottom compartment. Cells were treated with tirofiban (tiro), cangrelor or cilostazol (cilo) in the absence **(A)** or presence **(B)** of CCL5. n =4, median ± interquartile range. * $P<0.05$, Kruskal-Wallis with Dunn's test.
